# Dr. Samuel J. Dickey

**Published:** 1894-03

**Authors:** 


### Obituary.

### DR. SAMUEL J. DICKEY.

Died, on January 4, 1894, after a short illness, Dr. Samuel J. Dickey, aged seventy-four.

Dr. Dickey followed his professional duties from his early manhood, and consequently was one of the oldest in the practice of dentistry in Philadelphia.

He always had a deep interest in professional labor, but preferred, apparently, the quieter methods, as he took but a limited part in dental society work.

He was prominent in the Masonic Order and active as one of the managers of the Masonic Home, one of the valuable benevolent institutions of Philadelphia.

Dr. Dickey's genial nature gave him a large circle of friends and insured the respect of all with whom he was brought in contact.

He was buried with Masonic honors January 8, 1894.

